# Novel, Synchronous, First-person Perspective Virtual Simulations for Medical Students in Emergency Medicine

**DOI:** 10.5811/westjem.2022.11.57764

**Published:** 2023-01-10

**Authors:** Michael Sperandeo, Tiffany Moadel, Sezzy Yun, Stephanie Pollack, Michael Cassara

**Affiliations:** * Long Island Jewish Medical Center, Department of Emergency Medicine, New Hyde Park, New York; † Zucker School of Medicine at Hofstra/Northwell, Hempstead, New York; ‡ North Shore University Hospital, Department of Emergency Medicine, Manhasset, New York; § NYU Grossman School of Medicine, Ronald O. Perelman Department of Emergency Medicine, New York, New York

## BACKGROUND

Simulation is a mainstay in undergraduate and graduate medical education and is recognized as a “best practice” approach that is effective and complementary to medical education in patient care settings.^1^ Citing concerns for shortages in personal protective equipment and the need for social distancing during the peak of the coronavirus disease 2019 (COVID-19) pandemic,^2^ institutions compelled many medical student rotations and simulation centers to shut down.^3^,^4^ Educators were forced to pivot toward virtual learning methods.^5^,^6^ Due to social distancing precautions, students who were enrolled in our emergency medicine (EM) subinternship could not be physically present within the emergency department (ED) or simulation center. To address this education gap, we created a novel, synchronous, first-person virtual simulation experience.

## OBJECTIVES

Our program-level goals were as follows: a) to successfully implement a novel, synchronous, first-person perspective virtual simulation experience into an EM subinternship curriculum; and b) to assess the perceived efficacy of our novel, virtual simulation experience and virtual debriefing.

## CURRICULAR DESIGN

**Figure 1:**
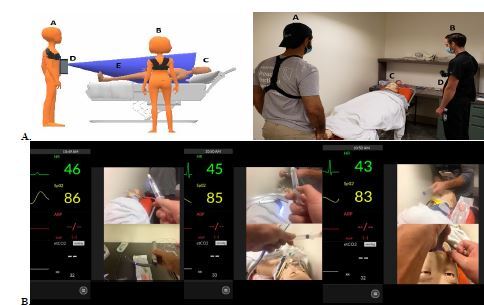
**A.** Conceptual framework for first-person perspective avatar (FPA) virtual simulation with body cameras. Key: (a, b) FPA; (c) patient mannequin; (d) body cameras. **B.** Learner’s view during endotracheal intubation.

Fourth-year medical students enrolled in our EM subinternship participate in a virtual simulation session during their rotation. Students access the session from a remote location. Sessions consist of two cases with groups of 2–4 students caring for a single virtual patient. The same two cases are uniformly used across all sessions. Sessions are hosted and broadcast over Zoom (Zoom Video Communications Inc., San Jose, CA). Students are told that they are working in a virtual ED and will be directing the care of an arriving patient. Two first-person perspective avatars (FPA)—facilitators equipped with body cameras—broadcast a first-person view of the virtual ED. The FPA bodycams function as the “eyes” of the learner, while the FPAs also function as the “hands” of the learner. All available medical equipment is visible through a video feed originating from the body cameras of the FPAs (Figure 1a).

Learners are encouraged to interact with the patient through the virtual environment.as they would in real life. Students receive a verbal hand-off from emergency medical services, including patient demographic information, the chief complaint, and a set of initial vital signs. A simulation operations specialist (SOS) plays the role of the patient. The SOS also facilitates the flow of the case and serves as a consultant. If a procedure is indicated, students must instruct the FPA in stepwise fashion how to perform the procedure for successful completion (Figure 1b). A dynamic vital signs monitor is displayed using the simulation training app Simpl.^7^ Case supplemental materials including laboratory and imaging results are uploaded onto the group chat on Zoom. Learners end the case by conducting a verbal hand-off to the appropriate medical service. The virtual simulation is followed by a virtual debriefing session led by trained EM simulation faculty using the PEARLS^9^ method.

Institutional review board (IRB) approval for this study was obtained at the Feinstein Insitutes for Medical Research at Northwell Health.. All learners are sent voluntary consent to participate in the research study. If consent is provided, students access a link to a single anonymous, post-experience survey using Google Docs (Google LLC, Mountain View, CA).

## IMPACT and EFFECTIVENESS

Since August 2020, we have piloted this experience to 76 fourth-year medical students. To date, 56 students responded to the post-experience survey (74%). Preliminary data on the perceived educational efficacy of this novel approach to virtual simulation has been largely positive: 95% of students found the experience beneficial, while 84% reported they learned more than they expected based upon their preconceived expectations. Prior to our virtual simulation experience, 72% of respondents felt virtual simulation could be an effective learning tool, compared to 98% after the session. Furthermore, 94% felt that virtual debriefing is equivalent to or more effective than in-person debriefing, suggesting that virtual debriefing is non-inferior to standard, in-person debriefing. Eighty-eight percent of students expressed they would participate in a virtual simulation experience again.

Select qualitative feedback from medical student participants included:

*With the residents wearing the body cams, we were really able to feel as if we were hands on.*

*I imagined something more conversational - like being presented with a case and talking through it but this was much more like a real in-person simulation.*

*In person is a better learning experience (more hands-on, more memorable, more active). However, I feel that virtual simulation is a super valuable tool. I feel that it should take the place of most didactics in the future. You learn way more from a simulation experience than from lectures. And because in-person simulations, although better, are not always feasible, I feel that that’s where virtual simulation can play a part in education.*

Informal faculty feedback applauded this simulation approach for its ease in deployment and perceived learner engagement. Drawbacks included the lack of fidelity in the virtual environment, inability for in-vivo, hands-on procedural training and technical difficulties related to a suboptimal internet connection. Study limitations include the fact that learners were only surveyed post-experience, a potential source of recency bias. Additionally, learners who enjoyed the experience may have been more likely to enroll in the study, a possible source of response bias.

In conclusion, we performed a pilot study implementing FPA virtual simulations for medical student rotators in EM. While students’ responses indicate a non-inferior level of perceived efficacy from FPA virtual simulations, additional study is required. Future directions require higher level Kirkpatrick evaluation of content retention. Assessment of actual learning as compared to perceived efficacy is paramount for further development of our virtual simulation experience.

We believe our conceptual framework has the potential in the post-COVID-19 era to bridge gaps in medical education in ways that were not previously possible. Medical students away from their home institutions can engage with faculty and peers in the virtual realm, opening new possibilities for formative and summative assessments. Our method can be applied internationally to introduce simulation-based medical education to lower resource settings without “brick and mortar” simulation infrastructure, fostering a new era of virtual collaboration and cooperation. We believe ongoing development of our novel method is critical in preparation for future challenges where pivots to virtual learning environments will be required.
